# Characterization of Water-Resistant Adhesive Prepared by Cross-Linking Reaction of Oxidized Starch with Lignin

**DOI:** 10.3390/polym17111545

**Published:** 2025-06-01

**Authors:** Chengyuan Liu, Huali Lin, Shichao Zhang, Hisham Essawy, Hongyan Wang, Longxu Wu, Xinyi Chen, Xiaojian Zhou, Antonios N. Papadopoulos, Antonio Pizzi, Ming Cao

**Affiliations:** 1 Yunnan Provincial Key Laboratory of Wood Adhesives and Glued Products, College of Material and Chemical Engineering, Southwest Forestry University, Kunming 650224, China; lcyuan0229@163.com (C.L.); wlx18786427724@163.com (L.W.);; 2 International Joint Research Center for Biomass Material, Southwest Forestry University, Ministry of Science and Technology, Kunming 650224, China; 3 Department of Polymers and Pigments, National Research Centre, Cairo 12622, Egypt; 4 Bamboo Research Institute of Zhejiang Academy of Forestry, Hangzhou 310023, China; 15990054143@163.com; 5 College of Forestry, Guizhou University, Guiyang 550025, China; 6 Department of Natural Environment and Climate Resilience, Democritus University of Thrace, Drama-Mikrochoriou, 66100 Drama, Greece; 7 Laboratoire d’Etude et Recherche sur le Matériau Bois (LERMAB), University of Lorraine, Cedex 9, 88000 Epinal, France; antonio.pizzi@univ-lorraine.fr

**Keywords:** lignin, starch, oxidation, cross-linking reaction, wood adhesive

## Abstract

Wood adhesives play a critical role in the wood processing industry; however, traditional formaldehyde-based adhesives pose health risks and are reliant on non-renewable resources. This study aims to develop a bio-based wood adhesive with excellent water resistance, focusing on environmentally friendly solutions. The synthesis of an oxidized starch-lignin (OSTL) composite adhesive was accomplished by modifying starch via oxidation and subsequent cross-linking with lignin. Ammonium persulfate (APS) was employed for oxidation of starch, introducing aldehyde groups that upgrade its reactivity with lignin. Subsequently, the oxidized starch (OST) was cross-linked with the phenolic rings of lignin, resulting in a strong network structure. The oxidation of starch and its cross-linking mechanism with lignin were investigated using the Fourier transform infrared (FT-IR), proton nuclear magnetic resonance (1H-NMR), and X-ray photoelectron spectroscopy (XPS) techniques, proving the formation of aldehyde and carboxyl groups with subsequent reaction possibilities. The effects of oxidant dosage, oxidation time, and the ratio of starch to lignin on the adhesive properties were systematically studied. The results demonstrated that the OSTL adhesive, prepared under optimized conditions, exhibited outstanding adhesion strength (1.23 MPa in dry state) and water resistance (0.94 MPa after 24 h cold water immersion, 1.04 MPa after 3 h in hot water, and 0.69 MPa after 3 h in boiling water), significantly outperforming conventional wood adhesives in terms of cold water, hot water, and boiling water resistance. In addition, the thermal behavior of the OSTL adhesive was further validated using differential scanning calorimetry (DSC) as well as thermogravimetric analysis (TGA). This study presents new insights and technical support for the development of green, environmentally friendly, and highly water-resistant lignin-based bio-adhesives.

## 1. Introduction

Wood adhesives play a crucial role in the wood processing industry. The most widely used wood adhesives are formaldehyde-based resin adhesives, including phenolic resin, urea-formaldehyde resin, and melamine-formaldehyde resin, which account for over 90% of the market share in the industry [1]. However, formaldehyde-based resin adhesives release free formaldehyde, which poses health risks, with another shortcoming being that their production relies on non-renewable petroleum resources. With the tightening of environmental regulations and the increase in consumers’ environmental awareness, eco-friendly biomass wood adhesives have developed rapidly [2]. Examples include lignin-based adhesives, tannin-based adhesives, and soy-protein-based adhesives.

Lignin is the second largest biomass resource in the world. The total annual production of natural lignin can reach 1.5 × 10^11^ tons, while the by-products of industrial lignin produced annually in the pulp and paper industries can reach 6 × 10^10^ tons [3]. However, the utilization rate of lignin is currently low, with only a small portion being effectively utilized. Lignin is a high-molecular-weight compound with a three-dimensional polyphenolic network structure, making it the largest biomass alternative to petroleum-based aromatic materials. Additionally, lignin plays a role in plant cell structures by linking cellulose and hemicellulose to support the cell wall, thus imparting certain adhesive properties and strength. Due to its structural characteristics, lignin has great potential as an adhesive [4]. However, lignin-based adhesives face challenges, such as poor water resistance, low bonding strength, and short storage life, which significantly limit their applications. Many researchers are using lignin either partially or entirely as a substitute for fossil-based materials to address the formaldehyde emissions associated with phenol-formaldehyde resins in adhesives. Sarkar et al. [5] used de-alkalized lignin to replace 50% of phenol in phenol-formaldehyde resins, resulting in lignin-modified phenol-formaldehyde resins (LPF), with adhesive performance reaching 78% of pure phenol-formaldehyde resin. Sudan et al. [6], after alkali-based phenolation modification of lignin extracted from black liquor, were able to replace 60% of phenol, producing high-performance LPF resin. Bornstein et al. [7] heated lignin extracted from sulfite pulp waste liquor with formaldehyde under alkaline conditions, then added a small amount of melamine to produce a water-resistant wood adhesive. The adhesive contained up to 70% lignosulfonate and significantly reduced the emission of free formaldehyde. These studies indeed demonstrated the strategy of using lignin to modify formaldehyde-based resin adhesives, which is not only more environmentally friendly and efficient but also established important foundations for the development of new bio-based adhesives. The use of lignin to modify formaldehyde-based adhesives can actually improve the bonding performance and reduce some formaldehyde emissions. However, it still requires the use of formaldehyde and does not fundamentally solve the formaldehyde emission problem. Therefore, many researchers explored the potential use of petroleum-based aldehydes, such as propionaldehyde [8], glutaraldehyde [9], and glyoxal [10], to replace formaldehyde. Comparatively, ethylene glycol has lower toxicity [11]. Also, using some non-toxic, green biomass aldehydes as alternatives to formaldehyde in adhesives’ preparation helps mitigate the issue of formaldehyde emission currently faced. Some studies focused on the use of lignin to replace a part of the phenol and then completely substituted formaldehyde with ethylene glycol in adhesives’ preparation. This approach resulted in adhesives exhibiting higher bonding performance than PF resin adhesives [12,13]. Furthermore, adding petroleum-based cross-linking agents to lignin-ethylene glycol resin was reported to enhance the adhesive’s bonding strength [7,8]. There are also studies [14] on adding hexamethylenetetramine as a hardener to lignin-ethylene glycol resin to cause an improvement of the adhesives’ dry shear strength up to 1.40 MPa. However, these cross-linking modifiers are expensive, leading to high production costs and limited economic benefits. Additionally, they still contain toxic substances. To address these issues, many groups opted to use furfural, lignin derivatives (such as hydroxybenzaldehydes, vanillin, syringaldehyde, and eugenol), as well as some non-toxic, green biomass aldehydes, like sugar aldehydes, all of which can substitute formaldehyde in adhesive formulations. Zhang [15] and Dongre [16], among others, utilized hydrolyzed lignin and hydroxymethyl furfural to prepare lignin-furfural adhesives, achieving a high yield of 85%. Its functional groups and curing mechanism are similar to phenol-formaldehyde resins. Moreover, it showed a larger molecular weight, broader molecular weight distribution, and higher glass transition temperature, storage modulus, and tensile strength compared to phenol-formaldehyde resins. Compared to PF adhesives, the curing of lignin-furfural adhesives required higher curing temperatures and long curing times. Therefore, lignin-furfural adhesives still cannot meet the industrial requirements in relation to the curing speed and temperature. Furthermore, to enhance the lignin activity, phenol pretreatment of lignin is sometimes an option, but this approach does not fully achieve the greenification of adhesives.

Natural green sugar-based materials, such as starch, sucrose, glucose, and cellulose, are widely utilized for their sustainability and high biodegradability, especially in the bio-materials field, serving as an excellent feedstock for sugar-based aldehydes. In a previous research work belonging to our group, an oxidized sucrose-lignin adhesive was prepared by achieving cross-linking between the oxidized sucrose with lignin. After immersing the adhesive-bonded three-layer plywood in hot water at 63 ± 3 °C for 3 h, the shear strength reached 1.42 MPa. In addition, the shear strength of the plywood samples after immersion in boiling water for 3 h achieved 1.03 MPa. This approach replaces formaldehyde with oxidized sucrose, addressing the issue of toxic formaldehyde emissions while maintaining excellent performance.

Starch is an abundant, inexpensive natural polymer [17,18,19,20]. However, it has low reactivity and typically requires chemical or physical modification for optimal application. It consists of two main components: amylose and amylopectin [21]. The ratio of amylose to amylopectin significantly affects the physical and chemical properties of starch and its applications [22]. Some studies explained that starch with higher amylose content often produces harder gels and more robust films [23]. Therefore, the current study will focus on utilization of soluble amylose-rich starch as a raw material for preparation of bio-aldehydes.

The high functionalization of starch is related to its abundant hydroxyl groups on the molecular chains of the sugar-based backbone, which can be easily chemically modified. Among suggested modifications, oxidation is one of the most common chemical treatments. After oxidation, the hydroxyl groups on starch molecules can directly upgrade to carbonyl or carboxyl groups along the molecular structure on the pretext that the hydroxyl groups at positions C2, C3, and C6 of the molecular structure of sugar-based materials are the most susceptible sites for oxidation agents to attack. Sugar-based materials can undergo oxidation using strong oxidizing agents, such as potassium permanganate, potassium dichromate, and nitric acid, to result in aldehyde and carboxylic acid compounds. However, this process can also cause significant structural damage of the sugars [24,25]. Persulfates are inexpensive and relatively mild oxidizing agents, which can break the glycosidic bonds in the starch molecular structures [26], leading to oxidation of the hydroxyl groups at positions 2 and 3 to aldehydes [18]. Furthermore, under heating conditions, persulfates can easily undergo hydrolysis in aqueous solutions to produce hydrogen peroxide and persulfate ions. They can also degrade the amorphous regions of lignin, hemicellulose, and cellulose, thereby facilitating the involvement of lignin macromolecular chains in many reactions. Compared to other oxidizing agents, persulfates offer greater advantages in lignin-based adhesives due to their environmental friendliness (non-toxic decomposition products), sustainability (derived from non-petroleum-based precursors), and efficient oxidation capacity under mild conditions [27]. Therefore, in this study, ammonium persulfate (APS) is chosen as the oxidizing agent to oxidize the active hydroxyl groups in starch molecules, aiming to obtain biomass aldehydes. The aldehyde groups can react with the active hydrogen atoms on the phenolic rings of lignin molecules to form lignin-based adhesives with excellent water resistance. Therefore, the Fourier transform infrared (FT-IR) and X-ray photoelectron spectroscopy (XPS) techniques are employed to confirm the formation of aldehyde groups in the oxidized starch products to ensure the liability for potential reactions between the oxidized starch and lignin. Finally, this study also investigates the effects of oxidation time, oxidizing agent dosage, and the starch-to-lignin mass ratio on the adhesive bonding performance of oxidized starch-lignin (OSTL).

## 2. Materials and Method

### 2.1. Materials

Poplar veneer with a moisture content of 8–10% was sourced from the local wood market. Sodium lignosulfonate was provided by Hefei Gansheng Biotechnology Co., Ltd., Hefei, China. Soluble starch was purchased from Shanghai Macklin Biochemical Co., Ltd., Shanghai, China. Ammonium persulfate (APS) was supplied by Tianjin Kemi Chemical Reagent Co., Ltd., Tianjin, China. Potassium bromide (KBr) was provided by Shanghai Aladdin Biochemical Technology Co., Ltd., Shanghai, China. The distilled water was prepared in the laboratory with a fully automatic distillation apparatus from Shanghai Xinyi Instrument Co., Ltd., Shanghai, China.

### 2.2. Preparation of Biodegradable Aldehyde via Oxidation of Starch

At a temperature of 60 °C, a certain mass ratio of starch was added into a three-neck flask equipped with a mechanical stirrer to prepare a 50 wt% aqueous solution of starch. After the starch was fully and evenly homogenized, a certain amount of APS ((NH_4_)_2_S_2_O_8_) as an oxidizing agent was charged and stirred for a certain period of time. After the reaction was complete, a filtered portion was exposed to freeze-drying to obtain the oxidized starch (OST) in powder form, while storing the remaining OST liquid in the refrigerator for subsequent analysis and use.

### 2.3. Preparation of OSTL Adhesive

A certain amount of lignin was added to the OST and stirred thoroughly to ensure that the solid content of the OSTL adhesive reached 50%. Then, the temperature of the water bath was raised to 90 °C and the reaction continued for 1 h. The reaction mixture was then cooled to room temperature to obtain the OSTL adhesive. In order to investigate the effect of other reaction variables, different oxidation times (0 h, 3 h, 6 h, 9 h, and 12 h) were employed. Furthermore, amounts of the oxidizing agent (9%, 11%, 13%, 15%, and 17% based on the starch mass) were varied while keeping a constant reaction time of 6 h at 60 °C to check the effect on the properties of the resulting OSTL adhesive. In addition, different mass ratios of the starch-to-lignin were set at 0.4, 0.6, 0.8, 1.0, and 1.2 to explore the effect on the properties of the adhesive.

### 2.4. Fourier Transform Infrared (FT-IR) Spectroscopy Investigation

The uncured adhesive sample was dried in a freeze-dryer, while the cured adhesive sample was dried in an oven at 200 °C, then each was mixed uniformly with potassium bromide in a 1:200 ratio and pressed into pellets. Then, the background interference was removed during scanning of the samples using a Nicolet 670 spectrometer over the wavenumber range of 4000 cm^−1^ to 400 cm^−1^ for a total of 32 scans with a resolution of 4 cm^−1^.

### 2.5. Proton Nuclear Magnetic Resonance (1H-NMR) Investigation

The freeze-dried adhesive was dissolved in deuterium oxide (D_2_O). The proton nuclear magnetic resonance (1H-NMR) spectrum was collected for the sample using an AVANCE NEO 500 spectrometer (Bruker Corporation, Zurich, Switzerland). The standard “zg3D” Bruker pulse sequence was applied for recording, while a frequency of 500 MHz was operated, with 65,536 data points collected over 16 scans. The relaxation delay was set to 3.28 s, and the chemical shifts were referenced relative to the deuterated solvent (D_2_O).

### 2.6. XPS Investigation

Using Al Kα excitation as a radiation source with an excitation energy of 1486.6 eV, the adhesive samples were examined before and after curing using the Thermo Scientific K-alpha XPS spectrometer. The model is Thermo Scientific K-Alpha, Waltham, MA, USA. Charge correction was applied with respect to the binding energy of C 1s at 284.8 eV.

### 2.7. Evaluation of Three-Layer Plywood Samples

**Figure 1:**
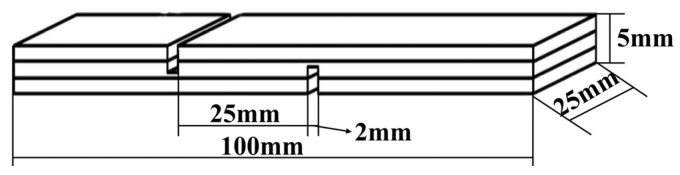
A schematic diagram revealing the dimensions used for three-ply plywood preparation for shear strength testing.

Three-layer plywood was prepared using poplar veneers with dimensions of 180 mm × 110 mm × 2 mm for each layer. The adhesive was applied on the surface of the specimen with a glue amount of 300 g/m^2^, and the pressing time was 10–15 min. After this, the plywood samples were pressed using a press machine that was purchased from Kunshan Rugong Precision Instrument Co., Ltd., Kunshan, China, at different hot-pressing temperatures: 160 °C, 170 °C, 180 °C, 190 °C, and 200 °C, with a unit pressure of 1 MPa, and a pressing time of 5 min. The obtained plywood samples were allowed to acclimate at room temperature for at least 24 h. Following this, the plywood samples were cut into standard test specimens, as shown in Figure 1, according to the testing requirements specified in the national standard GB/T 17657-2022 [28] for plywood bonding strength. According to the national standard GB/T 17657-2022, the testing for dry shear strength, 24 h cold water soaking strength (20 ± 3 °C), and 3 h hot water soaking strength (63 ± 3 °C) was conducted to assess the plywood’s resistance to boiling water. An additional 3 h of testing the soaking strength was performed. The values for each set of bonding strength evaluation were the averages of 6 measurements.

### 2.8. Estimation of the Hydrolysis Residue Weight of the Cured Adhesive

After curing of the adhesive samples in a high-temperature oven at 200 ± 3 °C, the samples were ground into 100-mesh powder. The adhesive powder was wrapped using a filter paper and immersed in water at 63 ± 3 °C. After 3 h of hydrolysis, it was dried to constant weight in an oven at 120 ± 2 °C. The residual ratio of the adhesive based on the mass ratio before and after hydrolysis was calculated according to the following formula:

$$\mathrm{residue}\;\mathrm{ratio}=\frac{m_{2}}{\;m_{1}}\times 100\%,$$

where $m_{1}$ is the mass of the cured adhesive before immersion and $m_{2}$ is the mass of the cured adhesive after immersion.

### 2.9. Antifungal Performance of the Adhesive

The antifungal activity was evaluated following a method described in the literature [29], where the starch samples (raw, OST, and OSTL) were prepared in 50% solution, and each solution was placed in a petri dish at room temperature and 90% humidity. The samples were observed at different time intervals for any sign of fungal growth or degradation.

### 2.10. Differential Scanning Calorimetry (DSC) Investigation

The adhesive samples weighing approximately 4.5 ± 2 mg were tested using a NETZSCH DSC204F1 differential scanning calorimeter under a flowing nitrogen atmosphere from 35 °C to 250 °C at a heating rate of 10 °C/min.

### 2.11. Dynamic Mechanical Analysis (DMA) Investigation

Poplar veneer specimens were cut with the grain into small pieces measuring 50 mm × 10 mm × 2 mm each. The adhesive was applied evenly to one piece of wood, with a glue amount of 300 g/m^2^. Then, another piece of wood was placed on the top and the specimens were allowed to rest for 15 min. The specimens were tested in a three-point bending mode at a heating rate of 5 K/min, from 35 °C to 300 °C, with a frequency of 20 Hz and a dynamic force of 2 N using a DMA-242 analyzer, NETZSCH, Germany, and the results were recorded and processed using Proteus analysis software, the version number is 8.17.39395.

### 2.12. Thermogravimetric Analysis (TGA)

The cured OSTL adhesive was subjected to thermogravimetric analysis (TGA) using Netzsch STA 2500, TA Instruments, Germany. A temperature range from 30 °C to 800 °C was scanned at a heating rate of 10 K/min.

## 3. Results and Discussion

### 3.1. Structural Analysis of OST

**Figure 2:**
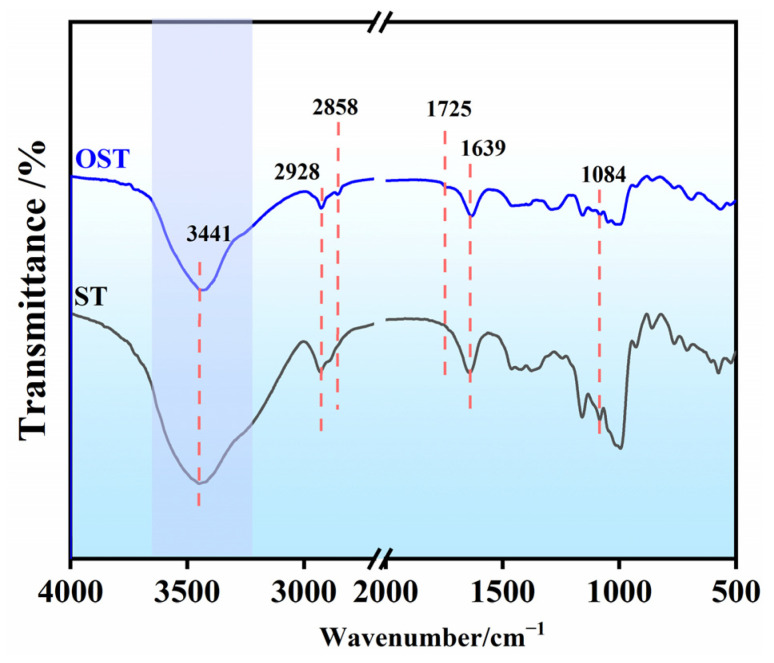
FT-IR spectra of starch (ST) and OST.

Figure 2 shows the FTIR spectra of ST and OST. The FT-IR spectrum of ST showed some characteristic features: a broad and intense absorption band around 3400–3500 cm^−1^ attributed to O-H stretching vibrations, peaks at 2928 cm^−1^ and 2850 cm^−1^ corresponding to -CH2- stretching vibrations, a peak at 1639 cm^–1^ indicating bending vibrations of hydroxyl groups in the starch molecular structure, and bands between 945 and 1209 cm^−1^ representing stretching vibrations of C-C, C-O, and C-O-C bonds [30]. On the other hand, the FT-IR spectrum of OST revealed emergence of a new peak at 1725 cm^−1^ attributed to the stretching vibration of (C=O) groups resulting from the oxidation. This indicated the formation of aldehyde or carboxyl groups after oxidation. Additionally, the signals between 945 and 1209 cm^−1^ mitigated, suggesting molecular chain fragmentation and depolymerization of starch molecules during the oxidation process.

**Figure 3:**
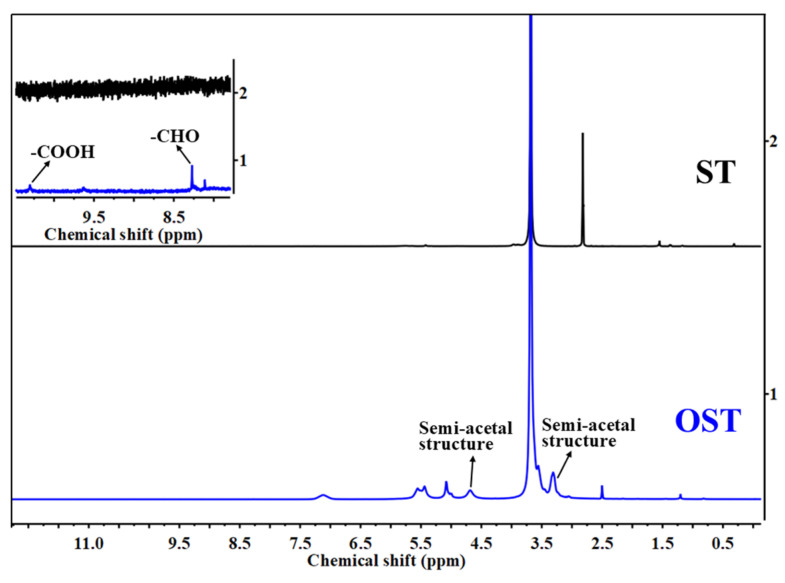
1H-NMR spectra of ST and OST.

To elucidate the products of OST in this study, 1H-NMR spectroscopic analysis was conducted on ST and OST. As shown in Figure 3, the 1H-NMR spectra of ST before and after oxidation revealed some structural differences. For OST, signals indicating aldehyde (CHO) groups appeared at 8.00–8.50 ppm, indicating successful oxidation of ST. According to previous studies [31], the C2–C3 bond of glucose units in the starch undergoes cleavage, resulting in the formation of two aldehyde units. However, the aldehyde signals in Figure 2 and Figure 3 are relatively weak. Some studies attributed this to an associated following formation of hemiacetal structures [32].

It was thus suggested that in this equilibrium system, there were aldehyde hydrates and hemiacetal groups, while free aldehydes were absent [31]. Furthermore, the high sensitivity of 1H-NMR spectroscopy enabled the detection of a relatively weak signal at 10.50 ppm, which presents evidence for carboxyl groups (COOH) formation. This also indicates that some hydroxyl groups in the ST structure encountered excessive oxidation to form carboxyl groups.

**Figure 4:**
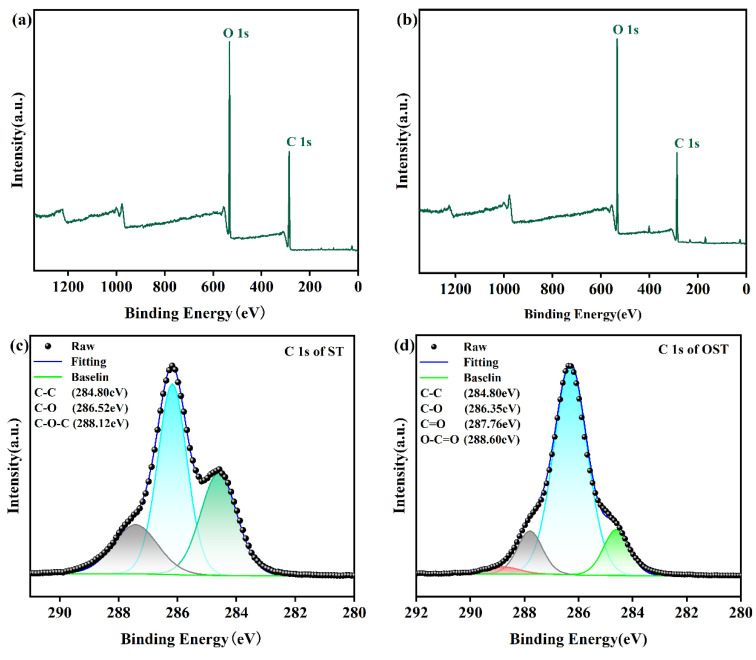
XPS spectra of ST and OST. (**a**) XPS full measurement spectra of ST, (**b**) XPS full measurement spectra of OST, (**c**) high-resolution C 1s spectrum of ST, and (**d**) high-resolution C 1s spectrum of OST.

**Figure 5:**
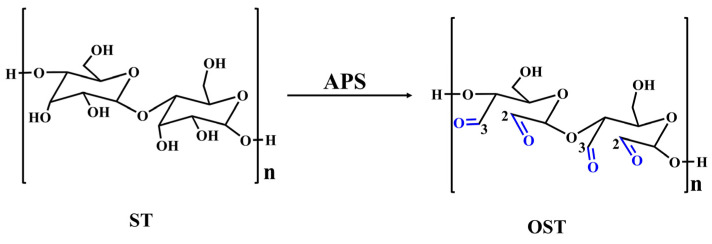
The process of oxidizing ST to generate OST using APS as an oxidizing agent.

To further validate the previous speculations, XPS analysis was conducted on ST and OST, as shown in Figure 4. Figure 4c displays the C 1s spectrum of ST, showing signals at 286.52 eV, attributed to C-O bonds, and at 288.12 eV attributed to C-O-C bonds. In comparison, the C 1s spectrum of OST revealed a signal peak at 287.76 eV, attributed to aldehyde groups, and another peak at 289.00 eV, referring to carboxyl groups. The elemental composition analysis of C 1s is collected in Table 1 and Table 2 for ST and OST, respectively, where it can be realized that the OST had a higher content of aldehyde groups, and only a small amount of ST may have been over-oxidized to form carboxylic acids. This is consistent with the obtained data from FT-IR and 1H-NMR. In summary, based on the FT-IR, 1H-NMR, and XPS spectroscopic analyses, a tentative structure of OST is depicted in Figure 5.

### 3.2. Structural Characterization of the OSTL Adhesive

**Figure 6:**
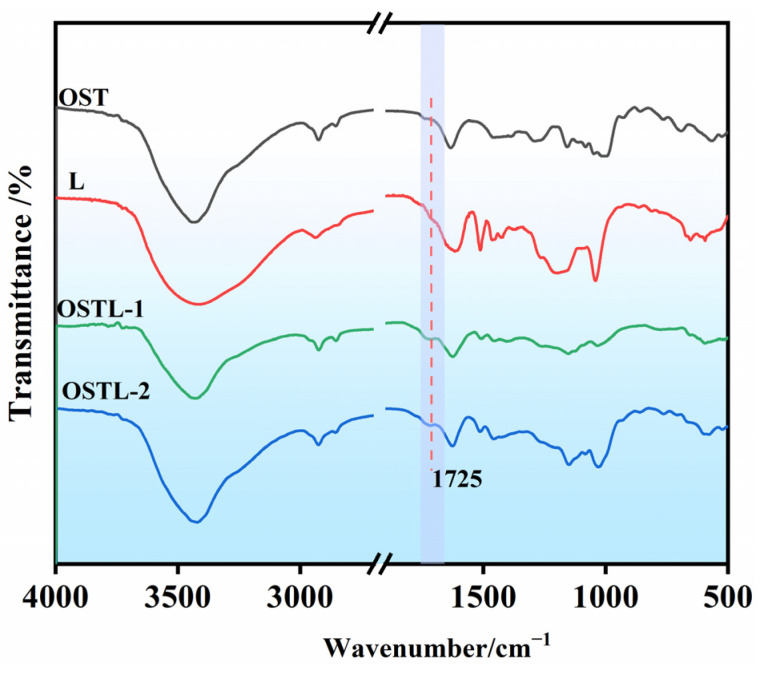
FT-IR spectra of OST, lignin (L), OSTL-1 (uncured resin adhesive), and OSTL-2 (cured resin adhesive).

The FT-IR spectra of OST, L, and the adhesive OSTL, before and after curing, are displayed in Figure 6. The peak at 1725 cm^−1^, attributed to carbonyl (C=O) groups, was present in OST, OSTL-1, and OSTL-2. No new peaks could be recognized in the adhesive before and after curing, whereas some slight shifts and a small change in peak intensities proved the curing was not significant, other than some bonds’ breakage and re-formation of another.

**Figure 7:**
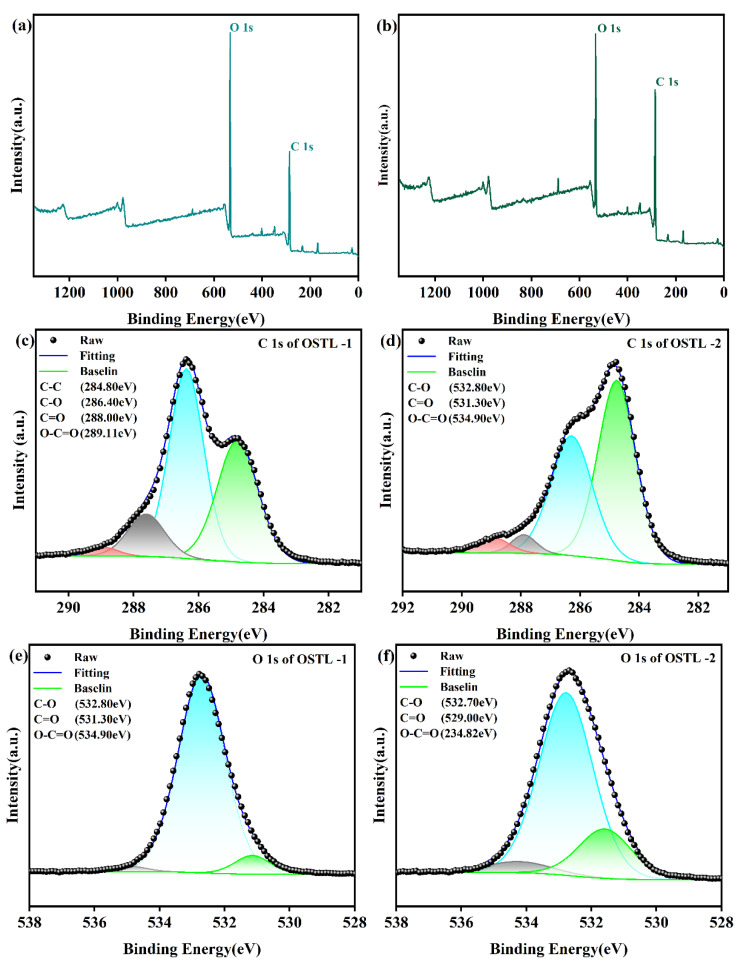
XPS spectra of OSTL-1 and OSTL-2. (**a**) XPS full measurement spectra of OSTL-1, (**b**) XPS full measurement spectra of OSTL-2, (**c**) high-resolution C 1s spectrum of OSTL-1, (**d**) high-resolution C 1s spectrum of OSTL-2, (**e**) high-resolution O 1s spectrum of OSTL-1, and (**f**) high-resolution O 1s spectrum of OSTL-2.

Therefore, to further investigate this, XPS analysis was accomplished on the adhesive samples before and after curing (Figure 7). From Figure 7, it is evident that there were no new functional groups formed in the adhesive before and after curing in the sense of chemical environments. However, XPS can quantitatively analyze the elemental content, as illustrated in Table 3 and Table 4. The change in the O 1s elemental content of the OSTL adhesive revealed that the content attributed to aldehyde groups at 531.30 eV decreased from 6.05% before curing to 0.14% after curing. This reduction was attributed to the consumption of aldehyde groups in reactions during curing, leading to a significant decrease in their content. Additionally, from Table 3 and Table 4, it can be observed that the content of O-C=O increased from 0.04% before curing to 0.24% after curing. This signifies that the present carboxyl groups formed due to over-oxidation can undergo esterification reactions with hydroxyl groups in L during curing, leading to an increase in the O-C=O content. Therefore, the reaction between OST and L involved an additional reaction between aldehyde groups and active sites in L, and the esterification of carboxyl groups derived from OST with hydroxyl groups on L. This can also extend to acetal or hemiacetal formation. These processes collectively promote the degree of cross-linking in the OSTL adhesive, leading to the formation of a robust cross-linked network structure, thereby enhancing the bond strength of the adhesive.

### 3.3. Evaluation of the Antifungal Performance of Adhesives

**Figure 8:**
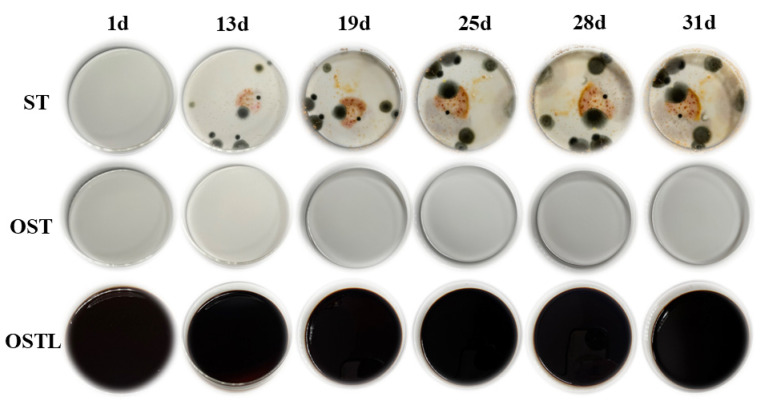
Mold resistance testing of ST, OST, and OSTL adhesives in liquid form.

Starch, a polysaccharide composed of anhydroglucose units, ranks as the second most abundant carbohydrate in plants. It serves as a crucial carbon source for microbial growth and reproduction. However, starch is highly susceptible to fungal infection [29,33], which limits its industrial applications significantly. As can be shown in Figure 8, the antimicrobial properties of various liquid samples were examined over one month, under conditions of room temperature and 90% relative humidity. It is noteworthy that mold growth began to appear around the 13th day in starch-based materials, and as time progressed, the mold spread further, gradually covering the entire base liquid of the culture dish. In contrast, OST and the samples cross-linked with L did not show any mold erosion, indicating that OST indeed acquired certain antifungal properties [34,35]. Meanwhile, lignin possesses natural antimicrobial properties [36], enabling the prepared OSTL adhesive to largely resist fungal corrosion.

### 3.4. The Effect of Different Oxidation Times on the Performance of OSTL Adhesives

**Figure 9:**
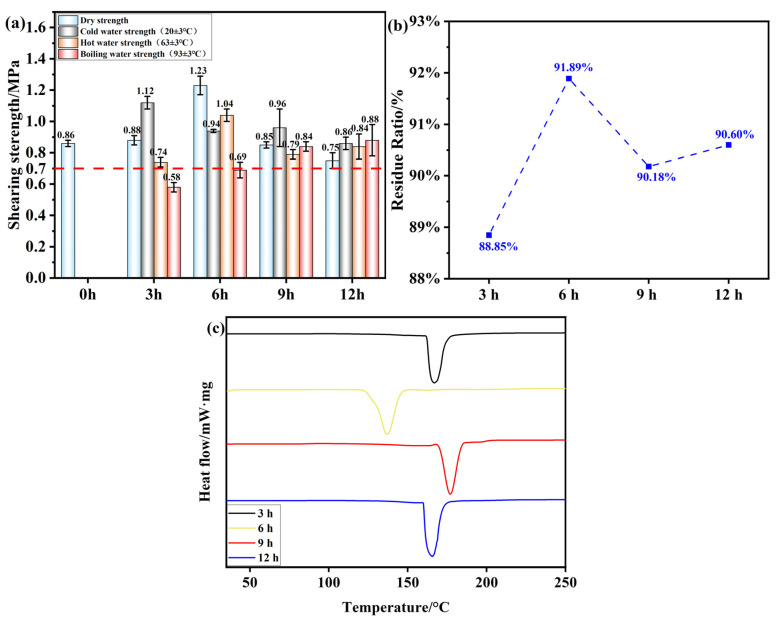
(**a**) Shear strength of plywood prepared using OSTL adhesive prepared under different oxidation times of OST as a precursor. (**b**) Hydrolysis residual rate of OSTL adhesive under different oxidation times of OST as a precursor. (**c**) DSC traces of OSTL adhesive prepared under different oxidation times of OST as a precursor.

Figure 9a illustrates the effect of different oxidation times on the adhesive performance of OSTL. In this experiment, the hot-pressing temperature was set at 200 °C with a pressure of 1 MPa and a duration of 5 min, while the adhesive formulation incorporated an oxidant addition of 13% and a ST-to-L mass ratio of 0.8. It is obvious from the figure that up to 6 h of oxidation time, both dry and wet shear strengths of the adhesives increased with prolongation of the oxidation times. For an oxidation time of 3 h, the shear strength of the OSTL adhesive-prepared plywood already met the standards for Class II plywood specified in GB/T 17657-2022. However, with further extension of the oxidation time, the hot water (63 ± 3 °C) shear strength of the adhesive decreased. This may present proof that with prolonged time, OST readily underwent aldol condensation with hydroxyl groups in the system, forming hemiacetals, thereby reducing the aldehyde content in the system. This may also be attributed to the over-oxidation, which can act in different ways to weaken the tendency of OST for a further reaction with L.

Additionally, to further verify the water resistance of the adhesive, this study also extended to testing the residual curing hydrolysis rate of the adhesive (Figure 9b). At an oxidation time of 3 h, the hydrolysis residual rate reached 88.85%. Increasing the oxidation time further to 6 h resulted in the highest residual rate of 91.89% for the adhesive. However, beyond 6 h of oxidation, most of the aldehyde groups in the OST were converted to the hemiacetal form. This resulted in insufficient aldehyde groups available for reaction with L, leading to insufficient cross-linking reactions and an unstable cross-linked network structure in the adhesive. Consequently, the adhesive became more susceptible to attack by water molecules, reducing its water resistance. This correlated well with the adhesive’s bonding performance results.

The bond strength and water resistance of adhesives are important criteria for evaluating the adhesive’s bonding performance. However, the overall performance of an adhesive cannot be solely decided from these parameters alone. Therefore, a thermal investigation was performed using differential scanning calorimetry, which is thought to reflect important information about the curing performance of the adhesives along with the thermal transition temperatures and heat flow relationships within the adhesive, assessing the absorption or release of energy as a function of temperature [37]. This included the OSTL adhesives prepared under different oxidation times of the OST as a precursor, as shown in Figure 9c. It can be observed that for an oxidation time of 6 h, the adhesive sample exhibited a distinct exothermic peak below 150 °C, whereas the exothermic peaks for the adhesives formulated with other oxidation time conditions occurred at temperatures higher than 150 °C (Table 5). This indicates that maximum reactivity could be attained for 6 h as the oxidation time of ST, and this assumption was supported by the fact that the adhesive acquired a lower apparent activation energy for curing. This also reflects that within a certain range of oxidation time, the highest level of aldehyde groups’ content can be achieved, leading to higher activity and lower heat required for curing reactions. This optimal level should also contribute to a more stable performance. Based on this, an oxidation time of 6 h was considered appropriate.

### 3.5. Effect of Oxidizing Agent Dose on the Performance of OSTL Adhesives

**Figure 10:**
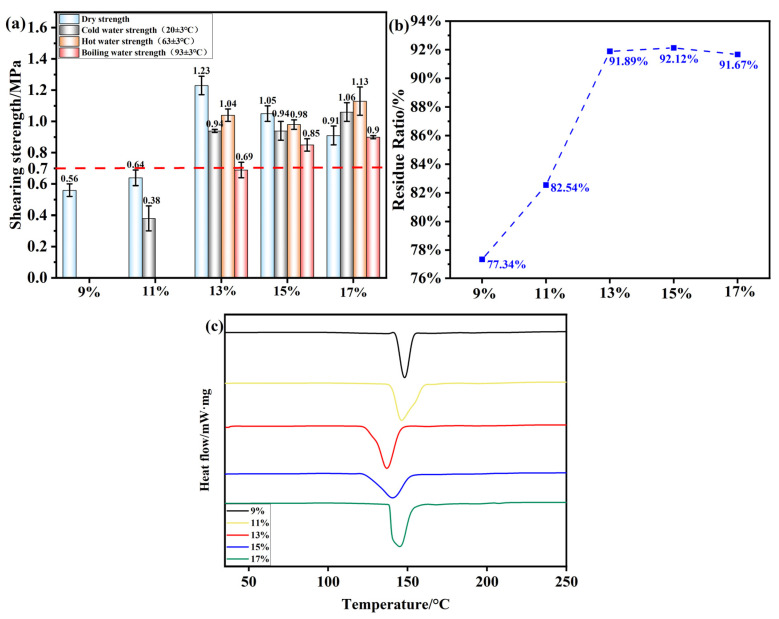
(**a**) Shear strength of plywood prepared using OSTL adhesives formulated with OST prepared using different oxidant additions. (**b**) Hydrolysis residual rate of OSTL adhesives formulated with OST prepared with different oxidant additions. (**c**) DSC curve of OSTL adhesives formulated with OST prepared using different oxidant additions.

The oxidant is an important reactant used in the modification of starch, directly affecting the degree of starch oxidation. Under hot-pressing conditions of 200 °C, 1 MPa, and 5 min, the effect of the oxidant addition amount on the shear strength of plywood prepared using OSTL adhesive is shown in Figure 10a, where the experimental conditions included an oxidation time of 6 h and a ST-to-L mass ratio of 0.8. From this, it can be observed that with an increasing amount of APS as an oxidant, the wet shear strength of plywood prepared using OSTL adhesive gradually increased. With an oxidant amount of 13%, the plywood’s hot water (63 ± 3 °C) shear strength reached 1.04 MPa, meeting the standards for Class II plywood specified in Chinese GB/T 17657-2022(0.7 MPa, the red dashed line in the Figure 10). As the oxidant APS gradually increased, although the wet shear strength of the plywood also increased, there was a drop in shear strength. This is simply justified by the fact that over-oxidation took place on the ST due to an excess of APS, leading to an insufficient level of aldehyde groups to undergo a good reaction with the L, whereas the large amount of formed COOH could cause some damage to the wood itself, resulting in reduced toughness and mechanical performance of the plywood.

This study also investigated the residual curing hydrolysis rate of an OSTL adhesive under different oxidant addition amounts, as shown in Figure 10b. Similarly, with an increase in the oxidant level, the residual curing hydrolysis rate significantly increased. When the adhesive reached a maximum residual rate of 92.12% at an oxidant addition of 15%, it subsequently decreased to 91.67% when the oxidant level exceeded 17%. This suggests that the adhesive’s water resistance did not directly correlate with increasing the oxidant amount for the range 13–17%.

Figure 10c reveals the conducted DSC study for the samples formulated with OST obtained under varying oxidant addition amounts while keeping other conditions constant. It can be seen that the temperatures of the obtained exothermic peaks during curing of the adhesive varied with the different oxidant addition amounts. This explains that the oxidant indeed played an important role in influencing the curing reaction of the adhesive. Table 6 reveals the curing temperatures and corresponding reaction enthalpy of the adhesive under different oxidant addition amounts. At 13% and 15% oxidant levels, the adhesive exhibited lower initial curing temperatures, but the peak curing temperature at 13% was lower compared to that at 15%, while the curing enthalpy at 13% was higher than that at 15%. Hence, an oxidant addition of 13% may be the most appropriate for formulations with L. Furthermore, from an economic perspective, adding a small amount of oxidant can achieve excellent bonding performance for the adhesive. Therefore, in this study, 13% was selected as the most suitable oxidant amount.

### 3.6. The Impact of Different Mass Ratios of ST-to-L on the Performance of OSTL Adhesives

**Figure 11:**
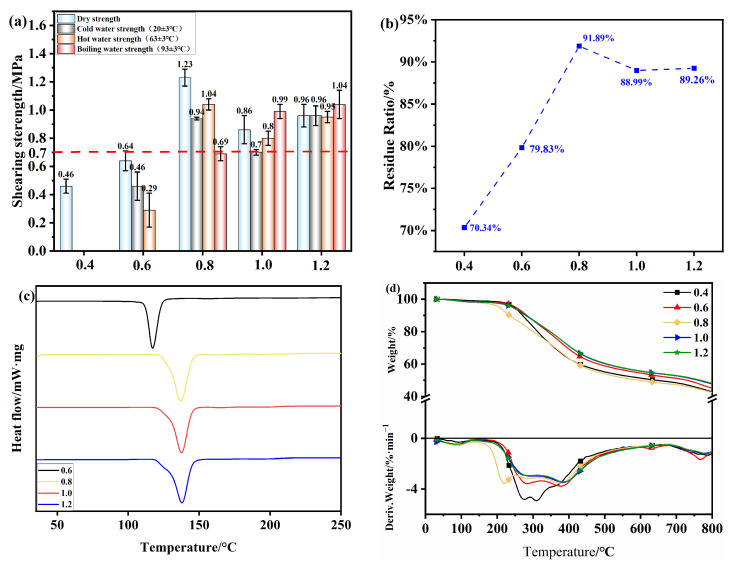
(**a**) Shear strength of plywood prepared using OSTL adhesives prepared with different ST-to-L mass ratios. (**b**) Hydrolysis residual rate of OSTL adhesives prepared with different ST-to-L mass ratios. (**c**) DSC thermograms of OSTL adhesives prepared with different ST-to-L mass ratios. (**d**) TGA traces of OSTL adhesives prepared with different ST-to-L mass ratios.

In order to further explore the influence of other factors on the bonding performance of OSTL adhesives, this study also investigated the performance variation of the adhesives prepared with different ST-to-L mass ratios. The experimental conditions included an oxidant addition of 13% and an oxidation time of 6 h, and the hot-pressing conditions were 200 °C, 1 MPa, and 5 min. As shown in Figure 11a, it can be observed that as the mass ratio of ST-to-L increased from 0.4 to 0.8, the dry shear strength of plywood prepared using OSTL adhesive also increased from 0.46 MPa to at least 1.23 MPa, achieving an increase of almost 3 times. This clearly demonstrates that increasing the amount of ST, under the same oxidant addition, likely provided more active aldehyde groups. This can effectively improve the bonding strength of lignin-based adhesives. It can be noticed that at a mass ratio of 0.8, the specimen’s hot water shear strength reached 1.04 MPa, showing a significant improvement. However, with the increase in the mass ratio, both the dry shear strength and wet shear strength of the specimens decreased. This could be because all active reaction sites on L already became involved in reactions. Increasing the ST content further may not effectively participate in cross-linking reactions with L. Additionally, excessive ST amounts may contain unoxidized hydroxyl groups, which are very hydrophilic and can reduce the water resistance of the adhesive. From Figure 11b, the residual hydrolysis rate of the adhesive prepared under different mass ratios confirmed this assumption: as the mass ratio increased, the adhesive’s residual rate initially increased significantly and then decreased.

Figure 11c,d depict the thermal performance of the adhesives prepared under different mass ratios. Figure 11c reveals the DSC traces of OSTL adhesives prepared under different mass ratios, complemented by the curing temperature and reaction enthalpy values (Table 7). As the mass ratio increased, the adhesive’s initial temperature of 110.5 °C was the lowest for the ratio 0.6, indicating an unmatured degree of cross-linking and less stable cross-linked network structure within the adhesive. This may correlate with the lower water resistance, consistent with the residual rate results of the adhesive. As the mass ratio continued to increase, especially at a ratio of 0.8, the adhesive exhibited the lowest initial curing temperature and highest enthalpy, indicating the reaction became more favored, leading to a deeper degree of curing. Therefore, at a ratio of 0.8, the adhesive may provide better performance, consistent with the results of the adhesive’s residual hydrolysis rate.

Figure 11d shows the TGA and relevant DTG traces of OSTL adhesives prepared under different mass ratios of ST-to-L. As seen from the figure, with the increase in temperature, all adhesive samples exhibited the same weight loss pattern. In the temperature range of 30–230 °C (Region I), a relatively low weight loss of about 10% was observed, which was likely due to the evaporation of small molecular substances and adsorbed/entrapped water [38]. In the temperature range of 230–500 °C (Region II), the primary phase of mass loss occurred, likely due to the breakdown of the adhesive’s cross-linked network and intensive degradation of the polymer chains within the molecular structure [39]. As the mass ratio increased, the initial decomposition temperature gradually increased. For example, the initial decomposition temperature was the lowest at a ratio of 0.4 and highest at a ratio of 1.2. This indicates that as the mass ratio increased, the higher aldehyde content effectively enhanced the degree of cross-linking between the chains, rendering the adhesive more resistant to thermal degradation.

Finally, based on the comprehensive analysis of adhesive bonding strength, residual hydrolysis rate, and thermal performance, it can be concluded that the adhesive exhibited optimal performance at a ST-to-L mass ratio of 0.8.

### 3.7. Effect of Different Hot-Pressing Temperatures on the Performance of OSTL Adhesives

**Figure 12:**
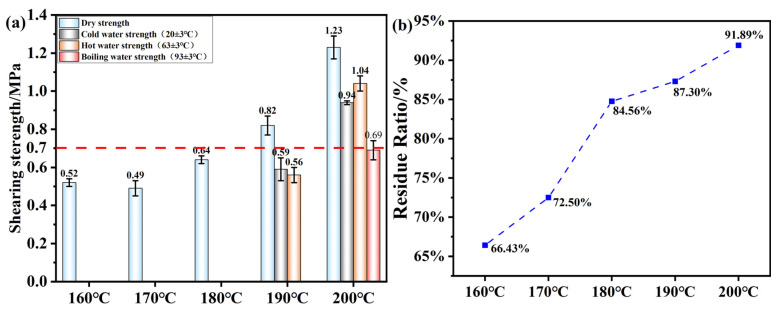
(**a**) Shear strength of plywood prepared using OSTL adhesives prepared at different hot-pressing temperatures. (**b**) Hydrolyzed residual rate of OSTL adhesives prepared at different hot-pressing temperatures.

Figure 12a shows the mechanical strength of the plywood bonded with OSTL adhesives prepared under different hot-pressing temperatures (1 MPa, 5 min). The adhesives were synthesized with 13% ammonium persulfate (APS, based on starch weight), a starch-to-lignin mass ratio of 0.8, and an oxidation time of 6 h. It can be observed that as the hot-pressing temperature increased, both the dry and wet shear strength of the adhesive gradually improved. Combining this with the analysis of the adhesive’s water resistance shown in Figure 12b, it can be realized that the reaction only occurred fully at higher temperatures. This may be because the L grade used in this study was industrial L, which had a higher degree of condensation and thus provided fewer active reaction sites. Starch, as a macromolecular polysaccharide, also required higher temperatures to undergo maximum cross-linking reaction between the two macromolecular reactants.

## 4. Conclusions

Oxidation of starch represents a good approach for obtaining biomass aldehyde compounds, which can then be reacted with lignin to prepare environmentally friendly biomass adhesives. The oxidation process should be carefully undertaken under good control to ensure a minimal level of over-oxidation, which is associated with carboxyl groups’ formation at the expense of the more active aldehyde groups. The aldehyde groups underwent additional reactions with the active sites on lignin, while the carboxyl groups formed due to over-oxidation underwent esterification with the hydroxyl groups in lignin. This process should be balanced in order to ensure robust cross-linked network structures by the adhesive curing and elevated bonding performance. The mechanical performance of the prepared plywood and residual hydrolysis rate testing explained that the conditions of 6 h as the oxidation time, an oxidant addition of 13% based on the starch mass, and a starch-to-lignin mass ratio of 0.8, with a hot-pressing temperature of 200 °C, ensured that the OSTL adhesive bore excellent bonding performance. The plywood prepared using the adhesive prepared under optimized conditions achieved a dry shear strength of 1.23 MPa, cold water (24 h) shear strength of 0.94 MPa, and hot water (63 ± 3 °C, 3 h) shear strength of 1.04 MPa, to adhere perfectly to the Chinese standard GB/T 17657-2022 for Grade II plywood. At this time, the boiling water of the plywood reached 0.69 MPa. This was also confirmed by the thermal analysis conducted using DSC and TGA investigations, which were in complete accordance with other data.

## Figures and Tables

**Table 1:** Elemental composition of C 1s in ST.

Name	Peak	FWHM (eV)	Area (P) CPS. eV	Atomic (%)
C-C	284.80	1.21	46,384.29	35.70
C-O	286.52	1.51	69,450.80	53.45
C-O-C	288.12	1.84	14,109.65	10.86

**Table 2:** Elemental composition of C 1s in OST.

Name	Peak	FWHM (eV)	Area (P) CPS. eV	Atomic (%)
C-C	284.80	1.34	23,135.67	16.07
C-O	286.35	1.38	98,516.11	68.44
C=O	287.76	1.21	16,834.70	11.70
O-C=O	288.60	1.48	5449.34	3.79

**Table 3:** Elemental composition of O 1s in OSTL-1.

Name	Peak	FWHM (eV)	Area (P) CPS. eV	Atomic (%)
C-O	532.80	1.85	212,974.74	93.92
C=O	531.30	1.29	13,710.86	6.05
O-C=O	534.90	1.30	86.48	0.04

**Table 4:** Elemental composition of O 1s in OSTL-2.

Name	Peak	FWHM (eV)	Area (P) CPS. eV	Atomic (%)
C-O	532.70	2.48	158,164.89	99.62
C=O	529.00	0.54	225.13	0.14
O-C=O	534.82	0.54	373.98	0.24

**Table 5:** Curing characteristics of OSTL adhesives formulated from OST prepared under different oxidation times.

Time/h	Starting Temperature/°C	Maximum Exothermic Peak/°C	Termination Temperature/°C	Enthalpy/(J·g^−1^)
3	162.0	166.8	174.2	951.7
6	128.1	137.0	145.1	951.4
9	169.7	176.9	184.5	1156.0
12	159.4	165.4	172.2	1024.0

**Table 6:** Curing characteristics of OSTL adhesives formulated with OST prepared using different oxidant additions.

Additions/%	Starting Temperature/°C	Maximum Exothermic Peak/°C	Termination Temperature/°C	Enthalpy/(J·g^−1^)
9	142.8	148.1	153.5	666
11	144.0	154.8	162.1	997.4
13	128.1	137.0	145.1	951.4
15	125.5	141.1	150.1	814.2
17	126.3	140.5	149.8	779.8

**Table 7:** Curing characteristics of OSTL adhesives prepared with different ST-to-L mass ratios.

ST	Starting Temperature/°C	Maximum Exothermic Peak/°C	Termination Temperature/°C	Enthalpy/(J·g^−1^)
0.6	110.5	127.5	137.9	689.2
0.8	128.1	137.0	145.1	988.2
1.0	128.3	137.8	144.9	970.2
1.2	128.7	138.1	145.1	978.0

## Data Availability

The original contributions presented in this study are included in the article. Further inquiries can be directed to the corresponding authors.

## References

[1] Li R.J., Gutierrez J., Chung Y.-L., Frank C.W., Billington S.L., Sattely E.S. (2018). A Lignin-Epoxy Resin Derived from Biomass as an Alternative to Formaldehyde-Based Wood Adhesives. Green Chem. R. Soc. Chem..

[2] Shi X., Gao S., Jin C., Zhang D., Lai C., Wang C., Chu F., Ragauskas A.J., Li M. (2023). A Facile Strategy to Fabricate a Lignin-Based Thermoset Alternative to Formaldehyde-Based Wood Adhesives. Green Chem. R. Soc. Chem..

[3] Balk M., Sofia P., Neffe A.T., Tirelli N. (2023). Lignin, the lignification process, and advanced, lignin-based materials. Int. J. Mol. Sci..

[4] Rath S., Pradhan D., Du H., Mohapatra S., Thatoi H. (2024). Transforming lignin into value-added products: Perspectives on lignin chemistry, lignin-based biocomposites, and pathways for augmenting ligninolytic enzyme production. Adv. Compos. Hybrid Mater..

[5] Sarkar S., Adhikari B. (2000). Lignin-Modified Phenolic Resin: Synthesis Optimization, Adhesive Strength, and Thermal Stability. J. Adhes. Sci. Technol..

[6] Nonaka Y., Tomita B., Hatano Y. (1997). Synthesis of Lignin/Epoxy Resins in Aqueous Systems and Their Properties. Holzforschung.

[7] El Mansouri N., Pizzi A., Salvado J. (2010). Lignin-Based Polycondensation Resins for Wood Adhesives. J. Appl. Polym. Sci..

[8] Mansouri H.R., Pizzi A. (2006). Urea–Formaldehyde–Propionaldehyde Physical Gelation Resins for Improved Swelling in Water. J. Appl. Polym. Sci..

[9] Wu Z., Xi X., Lei H., Du G. (2017). Soy-Based Adhesive Cross-Linked by Phenol-Formaldehyde-Glutaraldehyde. Polymers.

[10] El-Mansouri N., Pizzi A., Salvadó J. (2007). Lignin-based wood panel adhesives without formaldehyde. Holz. Roh. Werkst..

[11] Van Nieuwenhove I., Renders T., Lauwaert J., De Roo T., De Clercq J., Verberckmoes A. (2020). Biobased Resins Using Lignin and Glyoxal. ACS Sustain. Chem. Eng..

[12] Hussin M.H., Aziz A.A., Iqbal A., Ibrahim M.N.M., Abd Latif N.H. (2019). Development and Characterization Novel Bio-Adhesive for Wood Using Kenaf Core (Hibiscus Cannabinus) Lignin and Glyoxal. Int. J. Biol. Macromol..

[13] Huzyan H.I., Abdul Aziz A., Hussin M.H. (2021). Ecofriendly Wood Adhesives from Date Palm Fronds Lignin for Plywood. BioResources.

[14] Chupin L., Charrier B., Pizzi A., Perdomo A., Bouhtoury F.C.-E. (2015). Study of Thermal Durability Properties of Tannin-Lignosulfonate Adhesives. J. Therm. Anal. Calorim..

[15] Zhang Y., Yuan Z., Mahmood N., Huang S., Xu C. (2016). Sustainable Bio-Phenol-Hydroxymethylfurfural Resins Using Phenolated de-Polymerized Hydrolysis Lignin and Their Application in Bio-Composites. Ind. Crops Prod..

[16] Dongre P., Driscoll M., Amidon T., Bujanovic B. (2015). Lignin-Furfural Based Adhesives. Energies.

[17] Fadlallah S., Roy P.S., Garnier G., Saito K., Allais F. (2021). Are Lignin-Derived Monomers and Polymers Truly Sustainable? An in-Depth Green Metrics Calculations Approach. Green Chem..

[18] Diaz-Baca J.A., Fatehi P. (2024). Production and Characterization of Starch-Lignin Based Materials: A Review. Biotechnol. Adv..

[19] Ferreira-Filipe D.A., Paço A., Duarte A.C., Rocha-Santos T., Patrício Silva A.L. (2021). Are Biobased Plastics Green Alternatives?—A Critical Review. Int. J. Environ. Res. Public Health.

[20] Weiss M., Haufe J., Carus M., Brandão M., Bringezu S., Hermann B., Patel M.K. (2012). A Review of the Environmental Impacts of Biobased Materials. J. Ind. Ecol..

[21] Amagliani L., O’regan J., Kelly A.L., O’mahony J.A. (2016). Chemistry, Structure, Functionality and Applications of Rice Starch. J. Cereal Sci..

[22] Luchese C.L., Benelli P., Spada J.C., Tessaro I.C. (2018). Impact of the Starch Source on the Physicochemical Properties and Biodegradability of Different Starch-based Films. J. Appl. Polym. Sci..

[23] Van Hung P., Maeda T., Morita N. (2006). Waxy and High-Amylose Wheat Starches and Flours—Characteristics, Functionality and Application. Trends Food Sci. Technol..

[24] Kristiansen K.A., Potthast A., Christensen B.E. (2010). Periodate Oxidation of Polysaccharides for Modification of Chemical and Physical Properties. Carbohydr. Res..

[25] Vanier N.L., El Halal S.L.M., Dias A.R.G., da Rosa Zavareze E. (2017). Molecular Structure, Functionality and Applications of Oxidized Starches: A Review. J. Food Chem..

[26] Apriyanto A., Compart J., Fettke J. (2022). A Review of Starch, a Unique Biopolymer—Structure, Metabolism and in Planta Modifications. Plant Sci..

[27] Zhao Z.Q., Ouyang X.P. (2012). Effect of Oxidation on the Structures and Properties of Lignin. Adv. Mater. Res..

[28] (2022). Test methods of evaluating the properties of wood-based panels and surface decorated wood-based panels.

[29] Shao J., Li X., Liu T., Chen S., Gong S., Cao J., Zhou W., Li C., Li J. (2024). Multiple Function Hyperbranched Polysiloxane Nanoclusters for Controlling a Cross-Linking Structure to Convert Soy Meal into a Strong, Tough, and Multifunctional Adhesive. ACS Sustain. Chem. Eng..

[30] Lin Q., Pan J., Lin Q., Liu Q. (2013). Microwave Synthesis and Adsorption Performance of a Novel Crosslinked Starch Microsphere. J. Hazard. Mater..

[31] Li J., Du M., Din Z.U., Xu P., Chen L., Chen X., Wang Y., Cao Y., Zhuang K., Cai J. (2023). Multi-Scale Structure Characterization of Ozone Oxidized Waxy Rice Starch. Carbohydr. Polym..

[32] Serrero A., Trombotto S., Cassagnau P., Bayon Y., Gravagna P., Montanari S., David L. (2010). Polysaccharide Gels Based on Chitosan and Modified Starch: Structural Characterization and Linear Viscoelastic Behavior. Biomacromolecules.

[33] Lyu F., van der Poel A.F.B., Hendriks W.H., Thomas M. (2021). Particle Size Distribution of Hammer-Milled Maize and Soybean Meal, Its Nutrient Composition and in Vitro Digestion Characteristics. Anim. Feed. Sci. Technol..

[34] Song S., Gao P., Sun L., Kang D., Kongsted J., Poongavanam V., Zhan P., Liu X. (2021). Recent Developments in the Medicinal Chemistry of Single Boron Atom-Containing Compounds. Acta Pharm. Sin. B.

[35] Haghighi F.H., Mercurio M., Cerra S., Salamone T.A., Bianymotlagh R., Palocci C., Spica V.R., Fratoddi I. (2023). Surface Modification of TiO 2 Nanoparticles with Organic Molecules and Their Biological Applications. J. Mater. Chem. B.

[36] Nguyen N.Y., Luong H.V.T., Pham D.T., Cao L.N.H., Nguyen T.T., Le T.P. (2025). Drug-loaded Fe3O4/lignin nanoparticles to treat bacterial infections. Int. J. Biol. Macromol..

[37] Chen S., Shi S.Q., Zhou W., Li J. (2022). Developments in bio-based soy protein adhesives: A review. Macromol. Mater. Eng..

[38] Cao L., Pizzi A., Zhang Q., Tian H., Lei H., Xi X., Du G. (2022). Preparation and Characterization of a Novel Environment-Friendly Urea-Glyoxal Resin of Improved Bonding Performance. Eur. Polym. J..

[39] Xu Y., Xu Y., Han Y., Chen M., Zhang W., Gao Q., Li J. (2018). The Effect of Enzymolysis on Performance of Soy Protein-Based Adhesive. Molecules.

